# A Novel Innate Immune-Enhancement Strategy Combined with IVIG Rescues Mice from Fatal *Staphylococcus aureus* Septicemia

**DOI:** 10.1155/2011/725483

**Published:** 2011-11-16

**Authors:** Gowrisankar Rajam, Gabrielle M. Hammons, George M. Carlone, Jacquelyn S. Sampson, Edwin W. Ades

**Affiliations:** Division of Bacterial Diseases, Centers for Disease Control and Prevention, Atlanta, GA 30333, USA

## Abstract

*Staphylococcus aureus* (SA) is a major community-acquired pathogen. The emergence of drug-resistant strains like, methicillin-resistant SA (MRSA), poses stiff challenges to therapeutic intervention. Passive immune-therapy with specific antibodies is being actively examined to treat fulminant infections with limited success. In this study, we demonstrate that P4, a 28-amino acid peptide, derived from pneumococcal surface adhesin A along with pathogen-specific antibody (IVIG; P4 therapy) is successful in enhancing the opsonophagocytic killing (OPK) of *S. aureus in vitro*. We questioned if it is possible to expand P4 therapy to treat staphylococcal infections *in vivo*. P4 therapy in combination with IVIG rescued 7/10 morbidly ill *S. aureus*-infected mice while only 2/10 survived in the control group.

## 1. Introduction

*Staphylococcus aureus, *a gram-positive bacterium, is a commensal organism known to cause a wide range of hospital- and community-acquired infections. It is also recognized for immune evasion mechanisms and its ability to develop multi-antibiotic resistance. The burden of staphylococcal disease has increased worldwide with the emergence of community-acquired methicillin-resistant *Staphylococcus aureus* (CA-MRSA) [1, 2]. Incidence of *S. aureus* bacteremia in the United States ranges between 20 and 40 cases per 100,000 with the case fatality rates ranging from 19 to 24% [3]. CA-MRSA rates have doubled in USA from the year 2000 to 2006 [4]. The major threats are the increase in the drug resistance in *S. aureus*, the spread among community isolates, and the limited new drugs with demonstrable efficacy on the drug-resistant isolates [3]. To address these challenges, we need to develop new tools and/or to retune old tools with new techniques. Therapeutic antibodies (passive immunotherapy) which can enhance the host immune system's ability to overcome *S. aureus* infection are ideal candidates to be evaluated as alternatives to combating this infection [5].

P4, a 28-amino acid peptide derived from pneumococcal surface adhesin A, has enhanced *in vitro* opsonophagocytosis in the presence of pathogen-specific IgG and rescued mice from life-threatening pneumococcal infection (P4-therapy) [6–8]. Recently, we have shown that P4-therapy can also be used to rescue mice from serious secondary pneumococcal infection following H1N1 viral infection in mice [9]. We questioned if it is possible to use P4 therapy to treat staphylococcal infections *in vivo*?

## 2. Materials and Methods

### 2.1. Peptide Synthesis

The amino acid sequence of the peptide designated as P4 was previously described [10, 11]. P4 peptide with free N- and C-terminus were synthesized and lyophilized at CDC Core Facility, Atlanta, GA. P4 peptide used in this study was synthesized in an ACT (Advanced Chem Tech) 396 multiple peptide synthesizer by use of standard and modified fluorenylmethyloxycarbonyl (Fmoc) protocols [12–14] and analyzed for fidelity of synthesis based on the protocols previously described [15]. Lyophilized peptide was resuspended in diethylpyrocarbonate- (DEPC-) treated water, sonicated for 3 minutes for dissolution and stored at −70°C. We derived 2 peptides, P6 and/or P7 from P4 sequence, and these peptides had no activation effect on the eukaryotic cells [11]. These peptides were used as negative controls in all *in vitro* experiments.

### 2.2. Species-Specific Antibodies Used in This Study

*S. aureus*-specific polyclonal and monoclonal antibodies used in this study are as follows: (1) gammaglobulin (IVIG, Gamunex, Telecris, NC), a pooled human serum having reactivity with a wide range of pathogens including *S. aureus* [8], (2) a rabbit polyclonal (ab35194, Abcam, Cambridge, MA) directed towards the soluble and structural antigens of the *S. aureus*, (3) rabbit polyclonal IgG directed towards the clumping factor A, ClfA of *S. aureus*, PAbClfA (courtesy Inhibitex, Inc, Alpharetta, GA), and (4) humanized mouse monoclonal anti-ClfA IgG, HM904 (courtesy Inhibitex, Inc, Alpharetta, GA). Our *in vitro* experiment design involved direct comparison of changes in opsonophagocytic killing or uptake in the presence or absence of P4.

### 2.3. Opsonophagocytic Killing Assay

To assess the relevance of P4 therapy to treat *S. aureus *infections, we used an* in vitro* opsonophagocytic killing assay (OPKA) [16]. HL-60 promyelocytic leucocytes differentiated into granulocytic lineage or fresh human polymorphonuclear neutrophills (PMNs) were used as effector cells. *S. aureus* clinical isolates used in this study were kindly provided by Dr. Joe Patti and Dr. John Vernachio (Inhibitex, Inc, Alpharetta,GA). Colony blot was used to screen three different *S. aureus* strains, namely, *S. aureus* strainL, strainN, and strainFB for the presence of surface-exposed antigens that react with the polyclonal and monoclonal antibodies listed above. Based on the colony blot analysis (data not shown), *S. aureus* strainN was selected for OPKA. While all three *S. aureus* strains reacted with IVIG (Gamunex), *S. aureus* strainN was the only that reacted with all four antibodies. P4 peptide solution (100 *μ*g/mL) was added to the OPKA mixture at the pre-opsonization stage, and the control wells received 10 *μ*L DEPC water instead. Opsonophagocytic killing of *S. aureus* strainN in the presence of strain-specific antibodies and complement without P4 served as the control. Increase in opsonophagocytic killing of the bacterium when P4 was added to this reaction mixture (bacteria, antibody, and complement) was calculated and expressed as % increase over control. Several experimental controls were maintained that include bacteria alone, bacteria with either one of the OPKA components, (antibody, complement, HL60 cells), or an incomplete combination that lacks any of the listed components. None of these controls results in the killing of bacteria. Since the primary objective of this study is to demonstrate the effect of P4 on the opsonophagocytic killing of *S. aureus* strainN, these controls were not included in the figures.

### 2.4. Isolation of PMNs from Human Blood

Heparinized venous blood was obtained from the Emory Blood Donor Services, Atlanta, GA. The Leukocyte separation kit, Histopaque-1119 (Sigma, St. Louis, MO) was used to separate granulocytes from the blood. Granulocytes were separated from the blood according to the methodology recommended by the manufacturer.

### 2.5. Mouse Strains

Mice (*Mus musculus*), strain Swiss Webster (ND4-SW), were obtained from Charles River Laboratories (Wilimington, MA, USA). Mice used in this study were 6–10 weeks old. 

### 2.6. Intranasal Infection and P4 Therapy

Intranasal inoculation of mice with *S. aureus *strainN and P4 therapy was performed using protocols previously described, with minor modifications [6, 8]. Female Swiss Webster mice (Charles River Laboratories, Wilmington, MA) 6 to 10 weeks of age were used in this study. All experiments were approved by the Institutional Care and Use Committee (IACUC) and conducted according to the institutional ethical guidelines for animal experiments and safety guidelines. All experiments were repeated three times. Swiss-Webster mice (*n* = 50) were infected intranasally with *S. aureus *strainN (~1.1 × 10^7^ cells/mouse). Our initial attempts to develop an intravenous challenge model were unsuccessful due to the rapid pathogenesis. Hence, we selected an intranasal challenge model that provided a window between moribundity and rescue. At 15 hours after challenge, 40/50 mice were moribund (score 2). Moribund mice were divided into four groups (10/group) that included three control groups (no treatment, P4 only, and IVIG only) and P4 therapy group (P4 + IVIG). Treatment control groups received two doses of IVIG (i.p; 100 *μ*L/mouse) or P4 (i.v; 100 *μ*g/mouse) at 15 hours and 24 hours after infection. These time points were chosen based on pathogen-specific challenge and dosage optimization experiments. Similarly, P4 therapy group received 2 doses of IVIG (i.p; 100 *μ*L/mouse), followed 20 minutes later by P4 (i.v; 100 *μ*g/mouse). Treated and untreated animals were monitored for disease symptoms and survival. Due to IACUC limitations, we have only used IVIG for *in vivo* experiments.

### 2.7. Score of Moribund Characteristics

Mice were monitored and visually scored twice daily for moribund characteristics. Mice were ranked on a scale of 5 to 0; 5 = healthy, normal coat, skin, eyes, breathing and activity/movement; 4 = healthy, beginning to look sick, ruffled coat; 3 = sick, ruffled coat, decreased activity; 2 = very sick, ruffled coat, decreased activity, eye secretions; 1 = near death, ruffled coat, little/no activity, eye secretions, decreased breathing, hence euthanized; 0 = dead.

### 2.8. Statistics

All *in vitro* and *in vivo* experiments were performed in triplicate on three separate assay days unless specified otherwise. Number of moribund animals after the treatment was recorded till 80 hours and the data were computed for significant difference among various groups using *t*-test: paired two samples for means (MS Excel 2003). Kaplan-Meier analysis was applied to the survival data.

## 3. Results and Discussion

P4 peptide, a 28-amino acid putative binding domain of Pneumococcal surface adhesin A (PsaA), is a multilineage cellular activator [11]. Here we have demonstrated that this eukaryotic cellular activation potential in P4 peptide can be exploited for rescuing mice from serious staphylococcal infections.

**Figure 1:**
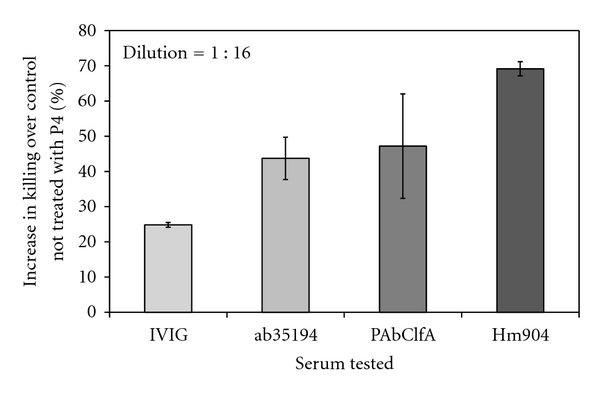
P4-mediated enhancement of *in vitro* opsonophagocytic killing of *Staphylococcus aureus *strainN. *In vitro* opsonophagocytosis killing assay was performed with HL60-cells-derived granulocytes. Addition of P4 increased the opsonophagocytic killing of *S. aureus *strainN in the presence of strain-specific antibodies. Maximum enhancement (≥70%) was seen with HM904 over control that had all OPKA components except P4 (*P* < 0.05). Antibodies used in this *in vitro* assay: Gamunex (IVIG), a pooled human serum having reactivity with a wide range of pathogens including *S. aureus*; ab35194, rabbit polyclonal directed towards the soluble and structural antigens of the *S. aureus*; PAbClfA, rabbit polyclonal IgG directed towards the clumping factor A, ClfA of *S. aureus*, and HM904, humanized mouse monoclonal anti-ClfA IgG.

**Figure 2:**
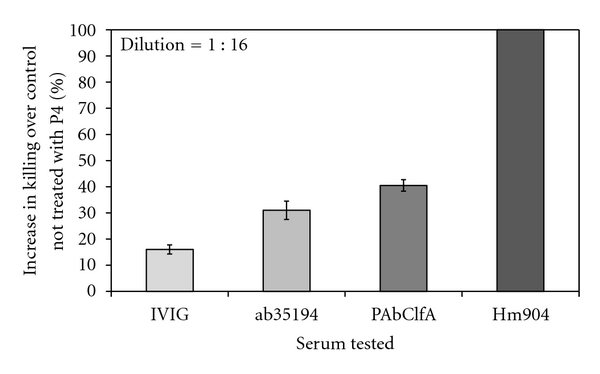
P4-mediated enhancement of OPK of *S. aureus* strainN with fresh PMNs isolated from human blood. *In vitro* opsonophagocytosis killing assay was performed with human peripheral blood PMN's. Addition of P4 increased the opsonophagocytic killing of *S. aureus *strainN in the presence of strain-specific antibodies. Maximum enhancement (≥100%) was seen with HM904 over control that had all OPKA components except P4 (*P* < 0.05). Antibodies used in this *in vitro* assay: Gamunex (IVIG), a pooled human serum having reactivity with a wide range of pathogens including *S. aureus*; ab35194, rabbit polyclonal directed towards the soluble and structural antigens of the *S. aureus*; PAbClfA, rabbit polyclonal IgG directed towards the clumping factor A, ClfA of *S. aureus*, and HM904, humanized mouse monoclonal anti-ClfA IgG.

The OPK assay demonstrates the presence of functional antibodies in sera [17]. Requirement of target-specific functional antibodies and complement supports the hypothesis that P4 enhances opsonophagocytosis of the target bacteria by the effector cells. P4 peptide has no direct effect on the bacterium and has no deleterious effect on human cells [11]. Additional experiments have shown that P4 peptide does not directly increase the intraphagocytic respiratory burst as seen with OxyBURST-labelled bacterium [8] or reactive oxygen species (ROS) production (data not shown). P4 enhanced the *in vitro* opsonophagocytic killing of *S. aureus* strainN in the presence of species-specific antibody and complement. The P4-mediated enhancement of OPK for *S. aureus* strainN was observed with HL-60-derived granulocytes and fresh PMNs from human peripheral blood (Figures 1 and 2). Among the antistaphylococcal antibodies, maximum enhancement in the opsonophagocytic killing of *S. aureus* strainN was seen with the humanized anti-ClfA MAb (HM904) with the fresh PMNs as the effector cells (100% with 6.6 *μ*g/mL of IgG, Figure 2). Previously, humanized anti-ClfA monoclonal antibody was shown to be effective as a prophylactic to protect lab animals from staphylococcal infection [5].

**Figure 3:**
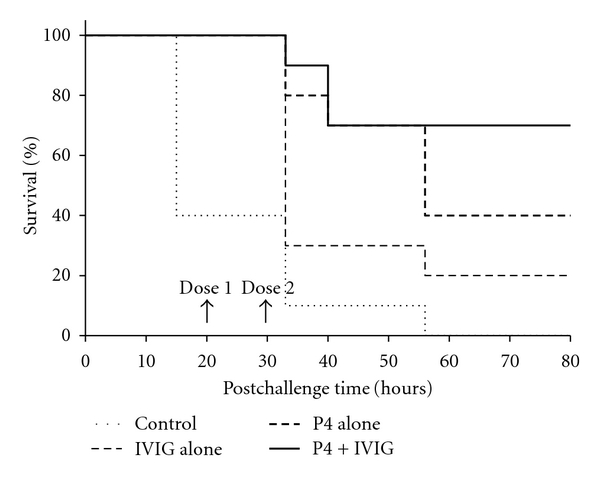
Survival of *S. aureus*-strainN-infected mice after P4-treatment. P4 with serotype-specific IgG confers protection to Swiss-Webster mice against intranasal *S. aureus* strainN challenge. Intravenous injection of P4 (100 *μ*g/mouse) with Gammaglobulin (100 *μ*L/mouse) at 15 and 24 hours after challenge provided highly significant protection (70%; *P* = 0.02) from *S. aureus* strainN infection (Kaplan-Meier analysis).

To ascertain if the P4-mediated enhancement of opsonophagocytosis would translate into therapeutic benefit, mouse rescue studies were carried out. Seventy percent (7/10) of mice that received P4 and IVIG therapy survived with complete remission of symptoms compared to untreated control group that had no survivors (*P* = 0.024, Figure 3). Even though there was a difference in the number of animals that survived in the single-treatment groups (P4 only = 40%; IVIG only = 20%), these differences were not statistically significant (*P* = 0.33). In this study, we have shown that P4-therapy combined with IVIG is effective in rescuing mice from severe *S. aureus* infection. The data suggest that P4 may be an important adjunct to immune therapy.

The P4-mediated rescue of mice is likely due to activation of circulating effector cells leading to an increase in the number of phagocytic cells participating in the opsonophagocytic clearance of the bacterial pathogen. We have hypothesized a mechanism of P4-therapy [18]; although the exact mechanism is still under investigation, previous studies suggest that P4 therapy augments innate immunity [9]. Polymorphonuclear neutrophils (PMNs) are the major innate immune component activated by P4 peptide as we have observed that mice treated with a neutrophil-depleting antibody, RB6-8C5, failed to respond to *in vivo* P4 therapy [7]. Recently, we have demonstrated that both altered trafficking and enhanced capacity of professional phagocytes are also associated with the potential protective mechanism for P4-therapy [9]. We have also observed rapid clearance of bacteria associated with decreased surface display of Fc*γ*RI and Fc*γ*RII on phagocytes [9]. In short, the presence of pathogen-specific antibody, effector cells, and complement are the critical factors that determine the effectiveness of P4 therapy against a particular pathogen [8].

## 4. Conclusions

*Staphylococcus aureus* continues to be a problem pathogen owing to its broad range of virulence factors and drug resistance. Passive immune-therapy (IVIG) with immune-enhancing agents like P4 peptide provides a viable alternative to treat *S. aureus*. Because of the high burden of staphylococcal disease and high case fatality rate, these findings merit further exploration.
